# Targeting TRIM5α in HIV Cure Strategies for the CRISPR-Cas9 Era

**DOI:** 10.3389/fimmu.2017.01616

**Published:** 2017-11-22

**Authors:** Daryl Anne Victoria Weatherley, Michael Terence Boswell, Sarah L. Rowland-Jones

**Affiliations:** ^1^Nuffield Department of Medicine, University of Oxford, Oxford, United Kingdom

**Keywords:** TRIM5α, HIV-1, HIV-2, PRYSPRY/B30.2, CRISPR-Cas9, adeno-associated virus, gene editing

## Abstract

In the past decade, studies of innate immune activity against HIV-1 and other retroviruses have revealed a powerful array of host factors that can attack the virus at various stages of its life cycle in human and primate cells, raising the prospect that these antiviral factors could be manipulated in immunotherapeutic strategies for HIV infection. This has not proved straightforward: while HIV accessory genes encode proteins that subvert or destroy many of these restriction factors, others, such as human TRIM5α show limited potency against HIV-1. However, HIV-1 is much more susceptible to simian versions of TRIM5α: could this information be translated into the development of an effective gene therapy for HIV infection? Reigniting research into the restriction factor TRIM5α in the era of superior gene editing technology such as CRISPR-Cas9 presents an exciting opportunity to revisit this prospect.

## Introduction

The HIV/AIDS epidemic continues to present a humanitarian crisis for the world’s most disadvantaged communities. Today, 36.9 million people are living with HIV, 70% of whom reside in sub-Saharan Africa. Antiretroviral therapy (ART) confers near-normal life expectancy on those adherent to the lifelong drug regimen. However, social and economic barriers to accessing care persist, and viral latency, drug toxicity and resistance contribute to long-term concerns for those on treatment. This means that there is a pressing need to achieve sustained virological remission in infected individuals.

TRIM5α restricts retroviral infection at an early post-entry stage in a species-specific manner through interaction of its PRYSPRY/B30.2 domain with the viral capsid (1). Human TRIM5α (huTRIM5α) has limited efficacy against HIV-1 *in vivo*, whereas the rhesus macaque TRIM5α and TRIM5-CypA fusion are highly effective against primate lentiviruses (2). huTRIM5α potently restricts another retrovirus, N-tropic murine leukemia virus (N-MLV) and appears to moderate HIV-2 infection, potentially contributing to an attenuated disease course (3, 4).

CRISPR-Cas9 technology is a powerful tool for editing small regions of the genome. It has proven superior to existing technologies exploiting targeted initiation of double-strand breaks including zinc finger nucleases (ZFNs), and transcription activator-like effector nucleases due to comparatively low levels of off-target mutagenesis and fast results (5, 6). Preclinical studies in humanized mouse models have shown that delivery of lentiviral vectors bearing hybrid TRIM5α isoforms leads to effective HIV-1 restriction; however, engineering HIV-1 resistance without the need for vectors that carry risks of immunogenicity and insertional mutagenesis would be a major advantage (7, 8).

## TRIM5α

TRIM5α is an interferon-inducible restriction factor of the tripartite motif family of proteins, which comprise over 70 members involved in various antiviral roles. The TRIMs feature a conserved set of domains: a RING domain, one or two B-boxes and a coiled-coil domain. They are most variable at the C-terminus responsible for viral capsid recognition, where 24 members possess a PRY/SPRY (SPRY) domain (9). TRIM5α is the most closely studied member of this family owing to the discovery of its antiretroviral role through expression screens of cDNA libraries from rhesus macaque cells (1). RNAi knockdown of peptidyl–prolyl cis–trans isomerase cyclophilin A in owl monkey cells yielded the discovery of another TRIM5 isoform that could potently restrict HIV-1, TRIMCyp-A (10). TRIM5α orthologs show significant interspecies variation in retroviral restrictive ability, which is thought to limit transmission of retroviral diseases between primates. Rhesus TRIM5α (rhTRIM5α) restricts HIV-1 and HIV-2 but does not restrict the closely related SIVmac, while huTRIM5α has a limited ability to restrict HIV-1 and SIVmac, but partially controls HIV-2 and potently restricts the gammavirus N-MLV (11).

The antiretroviral mechanisms of TRIM5α have not been fully characterized; however, multiple studies describe two steps. In the first step, TRIM5α specifically recognizes and assembles onto the viral capsid lattice in hexagonal nets (11, 12). Following this, TRIM5α induces abortive disassembly of the viral capsid core by accelerating the uncoating process before reverse transcription is complete, causing accumulation of reverse transcriptase products. This second step is dependent upon the RING domain E3 ubiquitin-ligase activity as the capsid-TRIM5α complex is targeted for proteasomal degradation (13, 14). TRIM5α also acts as a pattern-recognition receptor, and the restrictive ability of TRIM5α has been shown to be dependent on its ability to activate TAK-1-dependent innate immune signaling (11, 15). The capacity for TRIM5α to restrict HIV-1 appears to be dependent on cell type, TRIM5α restricts HIV-1 infection in Langerhans cells but not in other dendritic cells (16).

## TRIM5α Evolution

The SPRY domain has been the subject of positive selection and insertions/deletions associated with the divergence of New World monkeys from Old World monkeys and hominids (17). This is evident in the significant rates of nonsynonymous to synonymous change at this locus across 17 primate genomes encompassing 33 million years of evolution. Isolating the last 23 million years of primate evolution led to the identification of five residues within the protein under positive selection, falling within an 11–13 amino acid (aa) segment of the SPRY domain (the 13-aa “patch”) predicted to lie in coils at the protein–protein interface (17). Construction of chimeric proteins of human and rhesus orthologs showed that this patch was necessary and sufficient to confer measurable HIV-1 restriction, although not as effective as rhTRIM5α (17). Alteration of arginine 332 to proline or any uncharged residue (R332P) as the sole change in huTRIM5α was shown to potentiate restriction of HIV-1 (18, 19). The *Pan troglodytes* endogenous retrovirus (PtERV1), active 3–4 million years ago, was shown to be one of the likely culprits for this change as efficient restriction of chimeric PtERV was abrogated in the presence of a hominid R332Q mutation but restriction of HIV-1 was improved (20). Taken together, this points to a situation of evolutionary “trade-off,” where fixation of R332 in the human lineage conferred resistance to PtERV1 but in combination with other antiretroviral factors rendered us poorly suited to the challenge of HIV infection.

## TRIM5α and HIV-1 Disease Association Studies

Given the evolutionary history of TRIM5α, it was hypothesized that present-day variation in huTRIM5α proteins might underlie the spectrum of resistance to retroviral infection across the population (21). Results from several studies evaluating the effects of *TRIM5* polymorphisms are summarized in Table 1. Much of the published literature describes the relationship between HIV susceptibility and *TRIM5*, with less attention paid to the effects on disease outcomes in infected individuals. At least one large study has shown that in HIV-1 infection, *TRIM5* genotype has little to no impact on disease progression (22). The results described in Table 1 are often inconsistent. This probably represents the complementary effects of SNPs in *TRIM5*, linkage disequilibrium, and variation in regulatory regions (21). Furthermore, none of the described studies included the prevalence of HIV-1 capsid variants, such as the H87Q mutation, which may play an important role in determining disease outcomes (23). The importance of capsid sequences in determining sensitivity to TRIM5α has been further demonstrated by the increased sensitivity of gag associated CD8^+^ T cell escape mutants to TRIM5α, indicating cooperation between the innate and adaptive immune response (24).

**Table 1:** *TRIM5* polymorphisms and HIV disease associations.

	Genotype	TRIM5α domain affected	Cohort population	HIV disease association	Reference
1	H43Y	RING	Central and South American	Diminished ability of TRIM5α to restrict HIV replication	(25)
2	43Y homozygote	RING	Hans and Dai Chinese	Allele appears paradoxically to protect against HIV infection	(26)
3	G249D	Linker 2 region between coiled-coil and PRYSPRY domains	Japanese and Indian	Associated with increased susceptibility to HIV-1 infection	(27)
4	R136Q	Coiled coil	Kenyan	Protects against infection	(28)
5	R136Q	Coiled coil	European Americans	More frequent in HIV-infected population	(29)
6	H43-136Q haplotype	RING and coiled coil	North-East Brazil	Increased frequency in HIV uninfected controls	(30)
7	G110R	B-box	Japanese	Increased susceptibility to HIV infection	(31)

## TRIM5α and HIV-2 as a Model of Elite Control

While HIV-1 infection is globally distributed and continues to increase in numbers, HIV-2 is endemic to West Africa and appears to be declining in prevalence across the region. Intriguingly, for many infected people HIV-2 has an attenuated clinical course when compared to HIV-1. Approximately 35–40% of individuals infected with HIV-2 do not progress to AIDS and display a prolonged asymptomatic stage with low/undetectable viremia compatible with a normal lifespan (32). Could this “elite control” be attributed to enhanced retroviral restriction by TRIM5α?

Mutations in the HIV-2 capsid determine vulnerability to TRIM5α: this has been mapped to residues 119 or 120 of the capsid (p26), where the presence of proline confers increased sensitivity to huTRIM5α and alanine or glutamine increases resistance (4). Confirming the significance of P119 in virus–host interaction, individuals in a West African HIV-2 cohort with this variant showed better disease control evidenced by lower viral load. A pattern demonstrating the cumulative effects of P119, P159, and P178 conferring superior viral restriction was evident and was predicted to reduce p26 dimer binding energies resulting in a less stable viral core (33). This contributes to more efficient epitope production and presentation, leading to stronger gag-specific cytotoxic T lymphocyte responses (34). Reciprocally, the amino acid sequence TFP found at positions 339–341 in rhTRIM5α confers HIV-2 restrictive activity even in the absence of P119 or P120 (35).

## TRIM5α: A Good Candidate for CRISPR Gene Therapy?

### Preclinical Studies *In Vitro*

Several studies have demonstrated superior retroviral restriction by human cells transduced with *rhTRIM5α* (36), but precise manipulation of key residues that confer anti-HIV-1 properties is still highly effective and less immunogenic. Simultaneously targeting *CCR5* and *TRIM5α* has produced HIV-resistant CD133^+^ hematopoietic stem cells (HSCs) by shRNA silencing *CCR5* and *TRIM5α* site-directed mutagenesis (37). Macrophages derived from these transgenic HSCs restricted R5 and dual-tropic HIV-1. A library of *TRIM5α* variants generated by PCR-based random mutagenesis showed R332–R335 double mutants have restrictive efficacy superior to R332, which restricts HIV-1 in the order of 10- to 30-fold (19, 38). It was then reported that R332–R335 mutants restricted a wide variety of HIV-1 subtypes, including CTL escape variants, with high efficacy. This was observed under the influence of a weak promoter, reducing the risk of off-target mutagenesis (39).

### Humanized Mouse Models

Humanized mice have to some extent met the need for animal models that faithfully reproduce HIV biology *in vivo*, overcoming some of the limitations of SIV strains used in research, which are problematic when considering the species-specific restriction afforded by TRIM5α. In SCID-hu mouse model engrafted with HSCs expressing a human-rhesus TRIM5α ortholog, it was shown that transgenic cells differentiated into macrophages resistant to HIV-1 infection. Mature, developmentally normal T-cells harvested from thymic grafts injected with transduced HSCs displayed eightfold restriction of an X4-tropic strain of HIV-1 *ex vivo*. These cells had a survival advantage in a mixed population in culture. Greater than 99% expressed the transgene, suggesting therapeutic reconstitution of the T cell repertoire with only HIV-1 resistant cells by competition might be possible (40).

In the most recent study of this kind, humanized mice were engrafted with HSCs transduced with a third-generation self-inactivating lentiviral vector expressing three anti-HIV genes: chimeric TRIM5α, a CCR5 shRNA and a trans-activation response decoy to broaden anti-HIV coverage. The HSCs engrafted at a rate of 17.5% without notable cytotoxic effects and induced downregulation of CCR5 expression with modest expansion when challenged with R5 and X4-tropic viruses. Gene-modified cells showed a selective survival advantage when challenged with R5 and X4-tropic strains *in vivo*. The mechanism was proposed to be HIV-1 exerting selective pressure on the mixed population of HSCs and the killing of infected unprotected HSCs. While plasma viremia in all mice was still established through unprotected infected cells, normal CD4^+^ levels were maintained. The authors state that in future stem-cell therapies, reconstitution of the immune system with HSCs protected against HIV-1 (41) would rely on such a protocol being optimized with regards to transduction efficiency and *in vivo* engraftment of transgenic stem cells (42).

## How Could TRIM5α Become a Realistic Therapeutic Target in Light of Gene Editing?

The most significant advance in gene editing in recent years has been the development of the CRISPR-associated Cas system. Homology-directed repair is facilitated by a double-stranded DNA targeting construct for precise insertion of a desired sequence (43, 44). Screening Cas9 orthologs has yielded a smaller Cas9 derived from *Staphylococcus aureus* suitable for packaging in adeno-associated virus vectors along with regulatory elements, and for paired nickase applications (45, 46). The SaCas9 endonuclease has undergone evaluation in mice for future *in vivo* applications and did not produce more off-target effects than SpCas9 (47).

**Figure 1:**
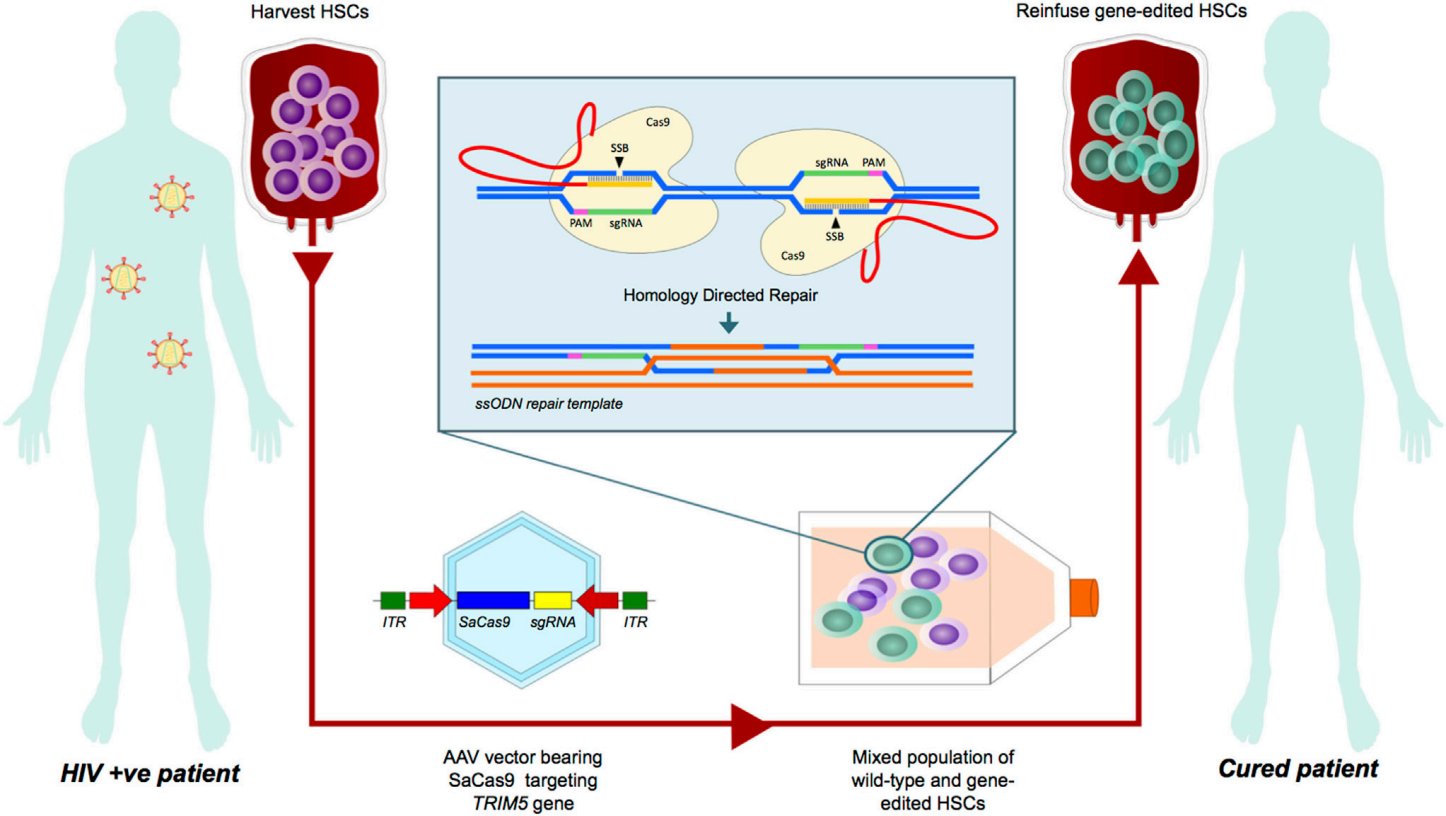
A theoretical model of TRIM5α gene therapy for HIV cure. This flow diagram demonstrates a theoretical model for *ex vivo* gene editing in hematopoietic stem cells (HSCs) to effect the R332P substitution using the newly described SaCRISPR-Cas9 system. HSCs harvested from an HIV-positive patient would be transduced with an adeno-associated virus (AAV) vector bearing the Cas9 apparatus, sgRNAs targeting *TRIM5*, and a repair template. A mixed population of HSCs would then be reinfused and among them, transgenic long-term repopulating HSCs would engraft, resulting in a durable subset of anti-HIV CD4^+^ T cells with a survival advantage in the face of viral challenge.

Using CRISPR-Cas9 with a repair template to effect the R332P substitution or other advantageous mutations in HSCs would be a first step in developing this strategy (see Figure 1). Modeling a *TRIM5α* gene therapy on the proof-of-concept study infusing autologous ZFN-engineered CD4^+^ T cells homozygous for *CCR5* Δ32 into HIV-infected patients might be a logical next step, as these studies demonstrated selective survival advantage of autologous CD4^+^ T cells detectable at 42 months in one patient (48, 49). This would make *TRIM5*α gene therapy a contemporary of several other strategies modifying host factors to endow HIV resistance. Recently, multiplex gene engineering using CRISPR-Cas9 to ablate *CCR5* and *CXCR4* in primary human CD4^+^ T cells has proven effective *in vitro*, providing protection against switching viral tropism (50). However, it is important to consider the risk of neurological complications of West Nile virus in *CCR5*-deficient individuals (51). Using CRISPR-Cas to disrupt both transcriptionally active and latent virus by targeting the HIV-1 long-terminal repeat (LTR), which caps both ends of the integrated proviral genome has also been reported but is limited by the clustering of escape mutations at the Cas9 cleavage site (52, 53). It was recently shown that intravenous administration of saCas9/quadruplex sgRNAs in an all-in-one adeno-associated viral vector could both excise integrated proviral DNA in humanized mice and block active HIV-1 replication in standard mice (54).

It has been suggested that translational CRISPR-Cas9 strategies may work in concert with existing ART regimens to address the latent reservoir when a suitable delivery method for establishing stable Cas9 and sgRNA expression is found (55). Profiling the off-target effects of CRISPR gene editing is already achievable (56) and a strategy aimed at reducing the off-target effects that result from long-term expression of Cas9 nuclease has been developed; delivery of pre-packed Cas9 within lentiviral particles expressing sgRNAs that facilitate gene editing in primary T cells offers a safer approach for HIV gene therapy, albeit with a 20% reduction in gene editing frequency (57). Furthermore, the search for a posttranslational regulator of Cas9 endonuclease has been fruitful and an “off switch” derived from bacteriophage proteins has been found to prevent unnecessary propagation of CRISPR-Cas9’s effects after its work is done (58).

## Potential Pitfalls and Strategies to Overcome Them

Preclinical studies have identified potent anti-HIV transgenes; however, a barrier to translating these findings lies in the generation of sufficient numbers of transgenic HSCs while maintaining their repopulating capacity. To address this, new protocols to optimize the process of *ex vivo* gene editing and expansion of HSCs are in development. Selecting CD34^+^CD38^−^ HSCs specifically contributing to long-term multilineage hematopoiesis, and shortening *ex vivo* culture time to 24 h has been suggested as a technical update for HSC therapies involving long-term expression of a transgene (59). Furthermore, the pyrimidoindole derivative UM71 was shown to stimulate and maintain the *ex vivo* expansion of HSCs for up to 7 days, potentially allowing production of therapeutic volumes of transgenic HSCs (59). Recently, it was shown that SCID-X1 mice could undergo lymphoid reconstitution with transgenic HSCs generated by homology-directed repair-mediated gene editing methods, including CRISPR-Cas9, following immunotoxin-based selective depletion of hematopoietic cells (60). This relatively mild conditioning regimen, thought to preserve tissue niches, was sufficient for reconstitution when at least 10% of functional HSCs engrafted (60). Furthermore, it was recently demonstrated that CRISPR-Cas9-mediated ablation of *CCR5* did not impact colony-forming potential in transgenic HSCs compared with control cells (61). *CCR5*-deficient long-term repopulating HSCs reconstituted multilineage hematopoiesis in mice, and following infection with a CCR5-tropic strain of HIV-1, transgenic CD4^+^ T cells showed a survival advantage (61). These proof-of-concept studies regarding the suitability of CRISPR-Cas9 for hematopoietic stem-cell therapies may represent a significant step forwards; however, it remains to be proven that transgenic HSCs could be safely translated to clinical use.

Any strategy aiming to introduce a stably expressed transgene *in vivo* will be beset with problems relating to immunogenicity. Several studies have shown efficient immune clearance of gene-engineered cells in the long term, even in severely immunocompromised patients (62–64). A potentially less immunogenic strategy might build on the finding that stabilized huTRIM5α is capable of HIV-1 restriction *in vitro* when expression is increased 20- to 30-fold (65). Small-molecule “performance-enhancing” therapies might present an alternative to gene editing with fewer associated risks; endogenous enhancers of TRIM5α antiviral activity include IFNα (66). Furthermore, the ability of HIV-1 to evade most antiretroviral strategies has been well documented in the case of pharmacological therapy (67) and it is unsurprising that anti-HIV transgenes have proved no exception. Both TRIMCyp- and TRIM5α-mediated restriction can be overcome by HIV-1 capsid mutations with little fitness cost to the virus (68, 69). However, the combined effect of HIV-1 capsid mutations, a gag-associated CTL response and TRIM5α may pressure capsid sequences to strains with reduced viral replicative capacity (70). The flexibility in this response should be further investigated and may offer an attractive alternative when compared to CCR5 ablation.

Persistence of transcriptionally inactive HIV in replication-competent latent reservoirs is the main barrier to development of a cure. Harbors of latent infection include the gut-associated lymphoid tissue and glial cells (71, 72). The “shock and kill” approach aims to reverse latency, then use combined ART and an engineered host immune response to clear the viral reservoir. The latency reversal agent SAHA in combination with ART effectively induced CD8^+^ T cell-mediated clearance *in vitro* (73, 74). However, there was no significant impact on either HIV DNA or quantitative viral outgrowth assay. A potential “shock” agent has been identified in the dCas9-synergistic activation mediator system for transcriptional activation at specified loci (75). This has been adapted for activation of the HIV-1 LTR in latent cells by targeting the enhancer of the LTR promoter, to provide the necessary “shock” using Cas9 depleted of nuclease activity resulting in the production of infectious virions (76–79). It remains to be seen whether this strategy can induce sufficient reactivation to purge the entirety of the latent reservoir and avoid reconstitution of the latent population by clonal expansion of cells harboring resistant mutants.

## Conclusion

Engineering an HIV-resistant immune system is emerging as a real possibility in the era of sophisticated gene engineering technology. Various host factors have been fielded as candidates for curative gene therapy but each has associated limitations, not limited to their multiple roles in immunity, switching viral tropism and the unparalleled ability of HIV to evade monotherapies by random mutagenesis. Though human versions of TRIM5α have very limited efficacy against the virus, simian TRIM5α orthologs fully restrict HIV, largely due to positive selection of a small number of specific residues localized to the C-terminal PRY/SPRY B30.2 domain. It is becoming easier to edit small regions of the genome to precise specification with minimal off-target mutagenesis; harnessing the simian TRIM5α template to confer superior HIV restriction capabilities on human cells will make TRIM5α a serious contender for the exciting gene therapies borne out of the CRISPR era.

## Author Contributions

DW wrote the first draft of the manuscript and drafted the final version with MB and SR-J. MB assisted in the drafting of the final manuscript. SR-J assisted in the drafting of the manuscript.

## Conflict of Interest Statement

The authors declare that the research was conducted in the absence of any commercial or financial relationships that could be construed as a potential conflict of interest.
